# Prevalence of Malaria and Associated Factors among Delivering Mothers in Northwest Ethiopia

**DOI:** 10.1155/2021/2754407

**Published:** 2021-12-07

**Authors:** Asmamaw Limenih, Woynshet Gelaye, Getaneh Alemu

**Affiliations:** ^1^Quarit District Health Office, Quarit, Amhara, Ethiopia; ^2^Department of Medical Laboratory Science, Bahir Dar University, Bahir Dar, Ethiopia

## Abstract

**Background:**

Malaria is one of the leading causes of morbidity and mortality especially in pregnant women and under-five-year-old children. However, data on the prevalence among delivering mothers, potential fetal transmission, and associated birth outcomes is lacking in Ethiopia.

**Objective:**

To assess the prevalence of *Plasmodium* infection from peripheral, placental, and cord blood samples among delivering mothers in Kuch health center, Northwest Ethiopia.

**Methods:**

An institution-based cross-sectional study was conducted among 218 delivering mothers from February to May 2021 in Kuch health center. Data on sociodemographic characteristics and clinical and obstetric history of mothers were collected using a structured questionnaire. Giemsa stained blood films from maternal capillary and placental and umbilical cord blood were examined for plasmodium infection. Data were analyzed using Statistical Package for the Social Sciences version 23 software package.

**Results:**

The prevalence of maternal, placental, and umbilical cord malaria was 6.4% (14/218), 2.3% (5/218), and 0.5% (1/218), respectively. *Plasmodium falciparum* and *Plasmodium vivax* accounted 3.7% (8/218) and 2.8% (6/218), respectively, in maternal peripheral blood but only *Plasmodium falciparum* was detected in placental and umbilical cord blood samples. Maternal malaria had significant association with primigravida (*χ*^2^ = 12.611, *p* = 0.002) and low birth weight (*χ*^2^ = 8.381, *p* = 0.004). Placental malaria was also significantly associated with low birth weight (*χ*^2^ = 32.255, *p* ≤ 0.001).

**Conclusion:**

The prevalence of malaria among delivering mothers was considerable. Maternal peripheral malaria had a significant association with gravidity and birth weight. Placental and umbilical cord malaria also had a significant association with birth weight. Pregnant mothers should be examined for malaria and receive appropriate treatment to prevent adverse birth outcomes.

## 1. Introduction

Malaria is a febrile illness caused by protozoan parasites of the genus *Plasmodium*. Five species of *Plasmodium*, *Plasmodium (P.) falciparum*, *P. vivax*, *P. malariae*, *P. ovale*, and *P. knowlesi*, are known to cause malaria. Plasmodium is transmitted by female Anopheles mosquitoes, which bite mainly between dusk and dawn. Malaria is a global public health problem that there were an estimated 229 million cases and 409,000 deaths in 87 malaria-endemic countries in 2019. In Africa, there were an estimated 215 million cases and 384,000 deaths in the same year [1]. Malaria affects all segments of the population; however, pregnant women and under-five-year-old children are at higher risk of infection and the parasites behave aggressively in these population groups [2, 3]. According to the World Health Organization report for the year 2019, around 12 million pregnant women were exposed to plasmodium infection in 33 moderate to high transmission countries in Africa, and 822, 000 neonates were borne with low birth weight (LBW) in those countries [1]. In order to reduce the health impact of malaria on pregnant women and their fetus and newborns, the World Health Organization has designed and recommended intermittent preventive treatment of malaria in pregnancy using sulphadoxine-pyrimethamine, especially in malaria-endemic regions [4]. However, this not implemented in Ethiopia so far.

Pregnant women and under-five-year-old children living in malaria-endemic areas are at risk of acquiring malaria depending on the degree of endemicity [5, 6]. In areas where malaria transmission is high and perennial, pregnant women are at risk of malaria infection mostly by *P. falciparum* [7]. On the other hand, infections are frequently asymptomatic, and therefore, many cases are undetected [2].

One of the main features of malaria in pregnancy is that women frequently presented with parasitized placenta during delivery [8]. This reveals that mothers who are infected during pregnancy have the probability to cause congenital malaria [4]. Since the placenta is affected by several mechanisms such as cellular adhesion, cytokine production, and mononuclear cell infiltration, malaria parasites can cross the placenta and infect the fetus [9, 10]. Particularly *P. falciparum* adheres to placental villi and reduces the placental blood flow to cause birth defects like LBW [2, 11, 12]. Malaria in pregnancy has many maternal and fetal complications like abortion, still birth, intrauterine growth restriction (IUGR), preterm delivery, LBW, and maternal death [2, 11–13].

Placental and congenital malaria are common in the first pregnancy and decline in consecutive pregnancies due to immunity development [5, 10]. Nulliparous and premiparous women are at higher risk of developing placental malaria [2, 11, 12]. Therefore, prevention of malaria before conception and early in pregnancy is essential to reduce the incidence of LBW [14]. However, the incidence and health impact of malaria is greatly affected by human, parasite, and environmental factors. This demands monitoring of the prevalence and associated morbidity at different geographical settings. Hence, the aim of the present study was to assess the prevalence of malaria among delivering mothers and associated birth outcomes in Northwest Ethiopia.

## 2. Materials and Methods

### 2.1. Study Design, Area, and Period

A facility-based cross-sectional study was conducted in Kuch Health Center from February to May 2021. Kuch is a subtown of Bure Zuria district in West Gojjam Zone, North Western part of Ethiopia. The area is located at geographical coordinates of 10° 29′ 00^″^ N and 37° 01′ 24^″^ E. There are six kebeles in the catchment of Kuch health center. The area has an altitude of 2038 m above sea level. The rainfall significantly varies with 80% of the annual rainfall occurs in June-August. The highest temperature occurs in January up to 32°C and the lowest in July and August reaching up to 19°C according to the woreda profile. According to data obtained from Bure Zuria district health office, the catchment population of Kuch health center in 2020/2021 is about 33,925 with 16,928 males and 16,997 females. From the total population, 10,558 live in urban and 23,367 live in rural areas. In the 2020/21 season, about 1143 pregnant women were estimated to give birth according to Bure Zuria Woreda health office. Malaria has minor and major transmission seasons in the study area, the major transmission season being from September to December while the minor transmission season is from April to June. However, despite the magnitude varies, malaria is transmitted throughout the year in the district.

### 2.2. Sample Size Determination and Sampling Technique

A single population proportion formula was used to calculate sample size (*n*) with the following assumptions: malaria prevalence (*p*) of 15.2% among delivering mothers according to a previous study from Wolkitie, Ethiopia [15], precision (*d*) of 0.05, and 95% confidence level (*Z*_*α*/2_ = 1.96).

(1) $$\begin{array}{c} n={\left(z_{\alpha /2}\right)}^{2}*p\frac{\left(1-p\right)}{d^{2}}={\left(1.96\right)}^{2}*\frac{\left(0.152*0.848\right)}{{\left(0.05\right)}^{2}}=198. \end{array}$$

After adding 10% (20) for nonrespondents, the final sample size was 218. Participants who fulfill the eligibility criteria were enrolled by convenient sampling technique. All delivering mothers in Kuch Health Center who were volunteer to participate were included in the study. Delivering mothers who took antimalarial or antibiotic treatment within a month before data collection, who were not permanently living in the catchment area of Kuch health center, who delivered twins, and those who were referred to hospital were excluded from the study.

### 2.3. Data Collection

#### 2.3.1. Questionnaire Data

Data on sociodemographic characteristics and clinical data were collected using a structured questionnaire in the labor ward at the postpartum period. The questionnaire was adapted from previous similar studies in Ethiopia [15]. Questionnaire data were collected from delivering mothers through face to face interview and by reviewing the antenatal care (ANC) follow-up log by trained midwives.

#### 2.3.2. Blood Sample Collection and Processing

Maternal capillary blood was collected, first by wiping the ring or middle finger with 70% alcohol swab and then piercing with sterile lancet. The first drop of blood was wiped away, and the second drop was used for thin and thick blood film preparation following standard protocol [16]. Immediately after delivery, the placenta was incised approximately 1.5 cm at the maternal side. Then, placental blood was aspirated by using a 5 ml syringe. Immediately, the blood was transferred to an ethylenediaminetetraacetic acid (EDTA) tube by midwives. In addition, just after the cord had been clamped, cord blood sample was obtained by wiping away excess blood from a clamped cord to avoid contamination with maternal blood. Then, about 2 ml of blood was taken from the umbilical vein [17] and transferred into an EDTA tube, thoroughly mixed by gentle inversion and taken to the laboratory for examination. Thin and thick blood films were prepared separately from maternal capillary blood, placental blood, and cord blood and processed by the Giemsa staining technique for the detection, identification, and quantification of malaria parasites following procedures explained elsewhere [15]. Blood samples from finger prick, placenta, and umbilical cord were collected by midwives while smear preparation, staining, and microscopic examination activities were conducted by laboratory technicians.

#### 2.3.3. Data Quality Control

Data collectors were trained about the data collection tools and the study procedure. Standard operating procedures were strictly followed during the laboratory test procedures. Each smear was examined by two laboratory technicians who are blind to each other's results. A malaria microscopy expert from the Amhara Public Health Institute reexamined slides with discrepant results among the two technicians. In such cases, results of the expert were reported as final results.

### 2.4. Statistical Analysis

Data was coded, entered, cleaned, and analyzed using statistical package for the social science (SPSS) version 23 software packages. Descriptive statistics was performed to describe study participants in terms of sociodemographic characteristics and clinical data. Prevalence of malaria in maternal peripheral, placental, and umbilical cord blood was also computed. Chi-square test was used to assess the association between malaria and sociodemographic and clinical factors.

## 3. Results

### 3.1. Sociodemographic Characteristics of Study Participants

A total of 218 study participants whose age ranged from 19 to 44 years old with a mean age of 29.4 and standard deviation (SD) of ±5.7 were included in the study. One hundred ten (50.5%) study participants were in the age group of 19-28 years old. Among 218 participants, 148 (67.9%) and 135 (61.9%) were farmers and participants who did not attend formal education, respectively (Table 1).

### 3.2. Prevalence of Malaria among Delivering Mothers

Among a total of 218 delivering mothers were screened; *Plasmodium* species were detected from peripheral blood of 14 (6.4%; 95% CI: 3.4-9.6) participants. Similarly, placental blood smears from 5 (2.3%; 95% CI: 0.5-4.6) participants were positive for *Plasmodium* infection. *Plasmodium* species was detected in umbilical cord blood of a single delivering mother (0.5%; 95% CI: 0.0-1.4). Maternal peripheral blood film examination revealed that *P. falciparum* and *P. vivax* monoinfections were detected in 8 (3.7%) and 6 (2.8%) participants, respectively. Regarding to parasite stages, only trophozoites were detected in blood film of 9 (4.1%) participants while in both trophozoites and gametocytes, and only gametocytes were detected in the blood film of 4 (1.8%) and 1 (0.5%) participants, respectively. *Plasmodium falciparum* was the only species detected in placental and cord blood smears. Out of five placental malaria detected, four had maternal malaria parasitemia. Only trophozoites were detected in 4 (4.1%) placental blood smears while both trophozoites and gametocytes were detected in 1 (0.5%) placental blood smear. The range of parasitemia in maternal peripheral and placental blood films was 480-4480 parasites/*μ*l and 200-1120 parasites/*μ*l, respectively. Cord blood malaria-positive blood films revealed a parasite load of 80parasites/*μ*l of blood. The mean maternal peripheral and placental parasite counts were 1290 parasites/*μ*l with SD of ±1196 parasites/*μ*l and 660 parasites/*μ*l with SD of ±487 parasites/*μ*l, respectively (Table 2).

### 3.3. Association of Malaria Parasitemia with Sociodemographic and Obstetric Characteristics

The prevalence of malaria was 78.6%, 14.3%, and 7.1% in primigravida, secundigravida, and multigravida participants, respectively, revealing a statistically significant association between malaria infection and gravidity (*χ*^2^ = 12.611, *p* = 0.002). Delivering mothers who had no full ANC follow-up were 36.2%, but there was no significant association between peripheral malaria and ANC follow-up (*χ*^2^ = 5.118, *p* = 0.077). Among the delivering mothers who gave LBW (<2.5 Kg) neonate, maternal peripheral malaria was detected in 27% of the mothers while 5.3% of malaria was detected among mothers who gave normal birth weight babies. There was a significant association between birth weight and peripheral blood malaria (*χ*^2^ = 8.381, *p* = 0.004). There was no significant association between previous malaria attack history and peripheral malaria (*χ*^2^ = 0.117, *p* = 0.732) (Table 3).

### 3.4. Association of Placental and Umbilical Cord Blood Malaria with Birth Weight

The prevalence of placental malaria was 27.3% and 0.97% in LBW and normal birth weight newborns, respectively. On the other hand, a single malaria case was found in the blood sample taken from the cord of a normal weighted baby. The chi-square test analysis showed that placental and umbilical cord malaria was significantly associated with birth weight (*χ*^2^ = 32.255, *p* ≤ 0.001) and (*χ*^2^ = 18.905, *p* ≤ 0.001), respectively (Table 4).

## 4. Discussion

In the present study, the prevalence of maternal peripheral malaria was 6.4%. It is comparable with a study result reported in Northwest Colombia where it was 9.1% [5], but it is higher than a study finding of 1.4% in eastern Uganda [18]. On the other hand, the present finding is lower than a study result of 15.2% in Wolkitie, Southern Ethiopia, [15] and 37.8% in Sudan [19]. Differences in malaria prevalence might be attributive to variation in season of data collection and geographical location. For example, data for the present study was collected in the dry season and the altitude of the data collection site ranges from 2020 to 2038 meters above sea level. This lowers malaria prevalence in the present study as compared to findings from Wolkite where the data was collected during the wet season, and the area is located at lower altitude 1910-1935 meters above sea level, both contributing for increased malaria transmission. Similarly, data from Sudan was also collected in the high transmission season [19].

The prevalence of placental malaria in this study was 2.3%. It is comparable to a study result in Wolkite (3.9%) [15] but it is lower than a study result reported in South Eastern Nigeria (57.6%) [3] and North Eastern Tanzania (8%) [20]. Out of 14 mothers who had peripheral malaria, 4 had placental malaria. *Plasmodium falciparum* was the only species detected in placental blood film, and it is relatively consistent with other studies [2, 11, 12]. This might be due to the fact that *P. falciparum* is the predominant species that adheres to placental villi, but *P. vivax* usually does not adhere to the placenta. The difference in placental malaria prevalence might be due to variations in diagnostic methods. In Tanzania, placental malaria was diagnosed using placental histology which is more sensitive than placental blood smear microscopy. The selection of study participants might be another reason that in Nigeria, only symptoms in mothers were enrolled in the study.

The prevalence of umbilical cord malaria was 0.5%. It was from a participant who had placental malaria but not peripheral malaria. This result is in agreement with a previous study conducted in eastern Uganda (0.95%) [18], but it is lower than findings from Nigeria (9.6%) [9] and Ethiopia (2.6%) [15]. Several factors including intensity of transmission, study population characteristics, use of preventive measures such as intermittent preventive treatment, insecticide-treated nets, and study design might justify this variation [21].

The prevalence of LBW in the present study (5%) was comparable to previous finding in Wolkitie Ethiopia which was 7.4% [15] and Nigeria 6.7% [22]. However, it was lower than results from Sudan (56.4%) [19], Papua New Guinea (14%) [23], and Tanzania (32%) [20]. The possible factors responsible for these variations might be acquired with immunity related to malaria transmission, nutritional status of the mothers, implementation of control activities, and coexisting clinical conditions.

In this study, gravidity had a significant association with maternal peripheral malaria (*χ*^2^ = 12.611, *p* = 0.002). It was similar to a result reported by Oringanje, in Cross River State in Nigeria in 2010 where primigravida mothers were more vulnerable to peripheral malaria [21]. Another study in Malawi also showed that primigravidity had a significant association with maternal peripheral malaria. This might be due to the fact that primigravida mothers are nonimmune. As gravidity increases, mothers become immune and become less vulnerable to malaria infection [24]. A meta-analysis in sub-Saharan Africa found that primigravidae mothers to have a higher prevalence of *P. falciparum* infection than women of higher gravidities which, in turn, higher risk of adverse birth outcomes. Immunity developed in multigravid women in their third or later pregnancy, but not in primigravidae, suggesting that variant-specific immune responses develop following exposure during pregnancy [25]. Variant surface antigen is expressed in pregnant women and binds to chondroitin sulfate A on the syncytiotrophoblasts lining the placental blood spaces. This variant surface antigen, termed VAR2CSA (Variant surface antigen 2-chondroitin sulfate A), is restricted to pregnant women. Primigravid women, who lack immunity to VAR2CSA, are highly susceptible to malaria. With each subsequent pregnancy, immunity builds, resulting in a lower risk of malarial infection in multigravidae. VAR2CSA-specific immunoglobulin G levels have been shown to inversely correlate with LBW and prematurity [2].

Birth weight had a significant association with maternal peripheral malaria (*χ*^2^ = 8.381, *p* = 0.004). There were significant associations between maternal history of malaria and low birth weight in previous studies [26–29]. The biologic plausibility and the consistency of these findings with previous reports all support the association between maternal malaria infection during pregnancy and LBW in the offspring. In the present study, placental malaria was significantly associated with birth weight (*χ*^2^ = 32.255, *p* ≤ 0.001). This is in line with a study result from Wolkitie, Southern Ethiopia, where placental malaria had a significant association with LBW [15]. Placental malaria was significantly associated with LBW in a meta-analysis result in sub-Saharan Africa. Infant mortality was three times higher for LBW babies than for those of normal weight in relation to malaria. LBW can be due to prematurity or IUGR. Identifying LBW cases caused by prematurity or IUGR can be difficult, as many African women are not certain of their gestational age. A previous analysis showed that a baby was twice more likely to be born with LBW if the mother had an infected placenta at delivery [25]. This is due to sequestration of *P. falciparum*-infected erythrocytes. Monocytes in the placenta induce altered cytokine profiles and complement activation that causes placental tissue injury and leads to placental insufficiency. Thus, it results in fetal growth restriction and LBW [30, 31]. Umbilical cord malaria was also significantly associated with birth weight (*χ*^2^ = 18.905, *p* ≤ 0.001). The result is in consistent with the study result in rural Malawi [32] and Bamenda, Cameroon [33]. However, it is difficult to give definitive conclusion on the association between placental and cord blood malaria parasitemia with birth weight because we our sample size was small.

In the present study, out of 5 participants who had placental malaria, one had both trophozoite and gametocyte stages. Even though trophozoites commonly sequester in the placenta [24, 34], previous studies also support the possible placental sequestration of gametocytes [35, 36]. In gestational malaria, when parasites adhere to the placenta, *Plasmodium falciparum* erythrocyte membrane protein 1 is the main adhesion receptor that adheres to the trophoblastic villous endothelium mainly through circumsporozoite antigen [37].

## 5. Conclusions

The prevalence of malaria among delivering mothers was considerable. Maternal peripheral malaria had a significant association with gravidity and low birth weight. Placental malaria and umbilical cord malaria also had a significant association with low birth weight. Hence, pregnant mothers living in endemic areas should be routinely screened for malaria and receive appropriate treatment to prevent adverse birth outcomes. As an alternative, intermittent preventive chemotherapy should be implemented in malaria endemic areas. We recommended further large-scale studies covering diverse geographical locations and using more sensitive molecular diagnostic methods.

## Figures and Tables

**Table 1:** Sociodemographic characteristics of delivering mothers in Kuch health center, Northwest Ethiopia, from February to May 2021 (*N* = 218).

Variables	Category	Frequency	Percent
Age	19-28	110	50.5
	29-38	90	41.3
	≥39	18	8.3
Marital status	Married	214	98.2
	Single	4	1.8
Educational status	No formal education	135	61.9
	Primary school	70	32.1
	Secondary school	10	4.6
	Higher education	3	1.4
Occupation	House wife	21	9.6
	Farmer	148	67.9
	Private business	37	17
	Government employee	12	5.5
Residence	Urban	97	44.5
	Rural	121	55.5

**Table 2:** Prevalence of *Plasmodium* infection in maternal, placental, and cord blood samples among delivering mothers in Kuch health center, Northwest Ethiopia, from February to May 2021(*N* = 218).

Source of blood sample	Number examined	Number of positives *n* (%)			Range of parasitemia/*μ*l of blood		
		*P. falciparum*	*P. vivax*	Total	*P. falciparum*	*P. vivax*	Total
Maternal peripheral blood	218	8 (3.7)	6 (2.8)	14 (6.4)	480-2480	520-4480	480-4480
Placental blood	218	5 (2.3)	—	5 (2.3)	200-1120	—	200-1120
Cord blood	218	1 (0.5)	—	1 (0.5)	—	—	

**Table 3:** Prevalence of malaria in relation to sociodemographic and obstetric characteristics among delivering mothers in Kuch health center, Northwest Ethiopia, from February to May 2021 (*N* = 218).

Variables category		Number examined	Malaria infection (%)	*χ* ^2^	*p* value
Age	19-28	110	8 (7.3)	0.267	0.875
	29-38	90	5 (5.6)		
	≥38	18	1 (5.6)		
Educational status	No formal education	135	8 (5.9)	0.535	0.911
	Primary school	70	5 (7.1)		
	Secondary school and above	13	1 (7.7)		
Occupation	Farmer	148	10 (6.8)	1.251	0.741
	House wife	21	2 (9.5)		
	Private business/government	49	2 (4.1)		
Residence	Rural	121	8 (6.6)	0.016	0.899
	Urban	97	6 (6.2)		
Gravidity	Primigravida	76	11 (14.5)	12.611	0.002
	Secundigravida	84	2 (2.4)		
	Multigravida	58	1 (1.7)		
ANC	No follow-up	24	4 (16.7)	5.118	0.077
	Partial follow-up (≤3 ANC)	115	7 (6)		
	Full follow-up (4 ANC)	79	3 (3.8)		
Previous preterm	Yes	7	1 (14.3)	0.744	0.388
	No	211	13 (6.2)		
Birth weight	<2.5 kg	11	3 (27.3)	8.381	0.004
	≥2.5 kg	207	11 (5.3)		
ABO blood group	AB	32	1 (3.1)	1.656	0.647
	A	51	5 (9.8)		
	B	56	3 (5.4)		
	O	79	5 (6.3)		
Previous malaria attack	Yes (within 1 year)	25	2 (8)	0.117	0.732
	No	193	12 (6.2)		
Malaria prevention method	Not used	74	7 (9.5)	2.920	0.404
	IRS	101	4 (3.9)		
	ITN	36	2 (5.6)		
	ITN and IRS	7	1 (14.3)		

**Table 4:** Placental and umbilical cord malaria in relation to birth weight among delivering mothers in Kuch health center, Northwest Ethiopia, from February to May 2021 (*N* = 218).

Variable	Number examined	Number positive	Birth outcome	*χ* ^2^ test	*p* value
Placental malaria	218	5	Birth weight	32.255	≤0.001
Umbilical cord malaria	218	1	Birth weight	18.905	≤0.001

## Data Availability

The original data for this study is available from the corresponding author's reasonable request.

## Abbreviations

ANC: Antenatal care
EDTA: Ethylenediaminetetraacetic acid
IUGR: Intrauterine growth restriction
LBW: Low birth weight.
